# Advancing longevity research through decentralized science

**DOI:** 10.3389/fragi.2024.1353272

**Published:** 2024-07-29

**Authors:** Maximilian Unfried

**Affiliations:** ^1^ Department of Biochemistry, Yong Loo Lin School of Medicine, National University of Singapore, Singapore, Singapore; ^2^ Healthy Longevity Translational Research Program, Yong Loo Lin School of Medicine, National University of Singapore, Singapore, Singapore

**Keywords:** decentralized science, longevity, aging, biotech, blockchain, crypto, open source

## Abstract

In an era marked by scientific stagnation, Decentralized Science (DeSci) challenges the inefficiencies of traditional funding and publishing systems. DeSci employs blockchain technology to address the misalignment of incentives in academic research, emphasizing transparency, rapid funding, and open-source principles. Centralized institutions have been linked to a deceleration of progress, which is acutely felt in the field of longevity science—a critical discipline as aging is the #1 risk factor for most diseases. DeSci proposes a transformative model where decentralized autonomous organizations (DAOs) facilitate community-driven funding, promoting high-risk, high-reward research. DeSci, particularly within longevity research, could catalyze a paradigm shift towards an equitable, efficient, and progressive scientific future.

## Introduction

Science has been a key engine of our prospering civilization. Yet the engine has become rusty as science is getting “less bang for its buck” (Bloom et al., 2020; Park et al., 2023). Despite having more professional scientists than ever, scientific progress and productivity across fields is decelerating, and research has become less innovative and disruptive (Bloom et al., 2020; Park et al., 2023).

This trend in decreasing productivity is evident in new drug development as well (Pammolli et al., 2011). Even though pharmaceutical R&D expenditure has consistently risen, the count of drugs securing approval for each billion US dollars allocated to R&D has decreased by around 50% every 9 years starting from 1950. This phenomenon has been coined Eroom’s Law–the reverse of Moore’s Law (Scannell et al., 2012; Ringel et al., 2020).

The academic establishment is also playing its role in slowing down productivity. In general, the academic workflow of grant writing, conducting research, scientific publishing and peer reviewing is expanding - creating ever more demands on researchers.

According to a survey related to the Covid Fast Grants program, an initiative that rapidly deployed $50 Million to Covid research, 50% of scientists spend 25%–50% of their time on writing grant applications, which leaves considerably less time to do research (COVID ‘Fast Grants’ sped up pandemic science, 2021; What We Learned Doing Fast, 2024). Moreover, evaluation of NIH grant proposals through peer review is not a good predictor of grant productivity, questioning the overall process (Fang et al., 2016). Furthermore, the typical R01 grant process is risk averse and slow, taking anywhere from 8 to 20 months to receive grant money for projects that mostly produce incremental progress (Illustrated Application and Grant Timelines. 2024).

Moreover, often conducted research results are unreliable. Science is in a reproducibility crisis, meaning that many scientific studies cannot be reproduced, creating a low signal to noise ratio (Baker, 2016). It is estimated that in preclinical research only 10%–25% of studies are reproducible (Begley and Ioannidis, 2015). While not being able to reproduce an experiment does not necessarily mean that the results were wrong, but that its results were not robust.

However, limited data availability and transparency in scientific publications contribute to the reproducibility crisis by obscuring the methodologies and datasets essential for validating experimental outcomes. This lack of open access to complete research data hampers other scientists’ ability to replicate studies, a cornerstone of scientific integrity, leading to a growing mistrust in published results and a questioning of the robustness of scientific research.

Moreover, the traditional academic publishing and peer review system faces criticism for its opacity, inefficiency, and susceptibility to bias (Kelly et al., 2014). Anonymity in peer review can lead to unaccountability, while the subscription model restricts access to scientific knowledge. The prevailing “publish or perish” ethos may prioritize quantity over impactful research, and the focus on high-impact journals can undervalue crucial replication studies. Consequently, the system often emphasizes prestige and journal impact factors at the expense of scientific rigor and the broader advancement of knowledge, impeding the very essence of scientific progress.

At its core academia is plagued by a misaligned incentive and reward system for scientists. It far too often incentivizes quantity of publications over quality, with scientists’ career progression often tethered to high-impact journal metrics, disincentivizing vital replication work and exploration of less popular but scientifically crucial inquiries. This skewed reward structure can divert attention from innovative and foundational research, as scientists vie for grants and tenure by aligning with short-term trends rather than pursuing diverse, risk-laden studies, and thereby stifling innovation. This academic climate, combined with low academic salaries has led to an exodus of biomedical researchers to industry (Watson, 2023).

The consequence of these combined issues stemming from centralized funding, centralized publishing and centralized institutions is resulting is a deceleration of scientific progress, bordering on scientific stagnation.

This crisis in the sciences is directly impacting the longevity field. While Longevity research is gaining traction, as of 2023, we currently have 0 drugs that are approved for humans to slow, stop or reversing the aging process. The only pharmacological longevity intervention that has consistently been shown to extend lifespan across animal species is rapamycin (Blagosklonny, 2019; Selvarani et al., 2021). While many compounds show lifespan extending effects in animals the data is in many cases non-comparable, ambiguous, and contradictory (Pabis et al., 2023; Spiridonova et al., 2023).

This is a problem because longevity science is seen as pivotal in addressing the demographic shift towards an aging population. It has the potential to extend healthspan, thereby reducing the socioeconomic burden of age-related diseases on healthcare systems (Olshansky et al., 2007; Scott et al., 2021). By unraveling the mechanisms of aging, longevity research aims to transform the quality of life for the elderly, ensuring that the years added to lifespan are matched by vitality and functional independence.

While the geroscience hypothesis targets aging directly, instead of the current paradigm of treating chronic age-related diseases (Kennedy et al., 2014), rapid progress in the longevity field is stifled by the same forces of the incumbent centralized institutions and their scientific dogmatism.

The decentralized science (DeSci) movement is the anti-thesis of the centralized scientific institutions that currently dominate academia. It is a startup to the incumbent big company. Decentralized Science aims at being open, transparent, and enforced through blockchain technology to create a better incentive system for scientists. Below DeSci will be explained in greater detail.

## What is decentralized science?

At the core of the Decentralized Science (DeSci) movement is the question: “How can blockchains coordinate humans to improve science and accelerate scientific progress?”.

A Blockchain is a type of distributed ledger technology where data is stored in blocks that are linked together in a chain. Each block contains a list of transactions, and once a block is filled, it is securely linked to the previous block (Justinia, 2019). This chain of blocks is distributed across a network of computers, making it decentralized. Hence, Blockchain technology enables secure, decentralized record-keeping and transactions without the need for a central authority or intermediary.

The emergence of Decentralized Science can be seen as the evolution of the Open Science movement, in that it merges values of open science with blockchain technology, such as smart contracts, as an algorithmic means to realize the open science values such as transparency, scrutiny, critique and reproducibility. Smart contracts are algorithmically governed protocols that autonomously execute contractual clauses encoded in digital code upon predetermined conditions being met. In other words, smart contracts are like automated digital agreements that execute themselves when certain conditions are met. As an example, in a laboratory rental agreement, a smart contract could automatically transfer rent from a tenant to a landlord each month, apply late fees if necessary, and record all transactions on a secure, transparent blockchain.

### Decentralized protocols for decentralized data storages

One early example of the value of blockchains is in data storage. Decentralized data storage mitigates risks associated with centralized data repositories, such as single points of failure or control by a single entity. In decentralized systems, data is distributed across a network, enhancing security and resilience against data loss or tampering. This ensures that once data is recorded, it cannot be altered without leaving a trace. This immutability guarantees the integrity of scientific data, making it reliable for future reference and analysis. Moreover, data storage protocols in DeSci enable transparent and traceable record-keeping. Every transaction or data entry is recorded on the blockchain, making it possible to track the history and provenance of data, which is crucial in scientific research for verifying results and reproducing experiments.

### Novel funding mechanisms for science

Various DeSci initiatives use blockchain technology to improve science funding. Generally, traditional funding bodies often favor conventional or less risky projects. DeSci models, by harnessing the power of community and broader investor bases, can provide avenues for funding high-risk, high-reward, or unconventional research projects that might not receive support through traditional channels. Moreover, instead of submitting long multi-page grant proposal that take months to be evaluated DeSci favors shorter grand proposals and a fast decision-making process.

For example, a Decentralized Autonomous Organization (DAO) is a novel bottom-up community-based organization model, without centralized control, which utilizes on-chain voting for decision making. In DeSci, DAOs can be used to govern and distribute funds for research. Community members, including scientists and funders, can have a say in decision-making processes, such as which projects to fund, based on their token holdings or other governance models. Through the on-chain voting and governance full transparency is assured as all votes are recorded and on the blockchain. In the DeSci space, DAOs are mainly centered around accelerating the progress of a specific science mission, through funding and promoting the respective field (DeFrancesco and Klevecz, 2022).

Another approach is to use cryptocurrency tokens to facilitate funding. Researchers can issue tokens representing a stake in their projects. This method democratizes investment in scientific research, allowing a broader base of small investors to contribute. Similar to traditional crowdfunding platforms, DeSci can enable the broader community, including the general public, to directly fund research projects they find compelling. This approach bypasses traditional grant-making institutions, reducing bureaucracy and potentially speeding up the funding process.

### Decentralizing publishing and peer review

In the landscape of scientific dissemination and evaluation, Decentralized Science has the potential to catalyze a paradigm shift, leveraging blockchain technology to reimagine traditional peer review and publishing structures. DeSci advocates for decentralized platforms for scientific publication, challenging the conventional dependence on centralized academic journals and fostering broader accessibility to research dissemination. This evolution is coupled with a commitment to open-access principles, ensuring transparency and the unrestricted availability of scientific findings. A novel aspect of DeSci is the incorporation of token-based incentives for peer reviewers, thereby acknowledging and valuing their pivotal contribution to the scientific process. This approach is complemented by a transition towards decentralized and transparent peer review mechanisms, which expand the spectrum of evaluative feedback and aim to diminish inherent biases. Such innovations are poised to accelerate the publication cycle, facilitate more robust data sharing practices, and enhance the reproducibility of scientific endeavors. Further, DeSci promotes the adoption of alternative metrics to evaluate research impact, diverging from traditional reliance on impact factors. In addition, it champions community-led governance models in scientific publishing, reinforcing research integrity and fostering a more inclusive, collaborative scientific community. This DeSci-driven transformation represents a significant leap towards a more equitable and efficient system for scientific communication and evaluation, in that researchers worldwide can publish at no or low cost and have equal access to scientific knowledge and the opportunity to contribute (Trovò and Massari, 2020).

### AI and decentralized clinical trials

The integration of Artificial Intelligence and Internet of Things with DeSci technologies has the potential to further enrich data analysis and real-time data collection, particularly in fields like environmental monitoring and precision medicine. Additionally, DeSci could revolutionize clinical trials, making them more decentralized (allowing more patients to participate), and efficient, potentially accelerating medical advancements. This shift should necessitate an evolution in ethical considerations and regulatory frameworks, ultimately creating a more open, collaborative, and equitable scientific ecosystem.

For decentralized clinical trials to gain recognition it is of utmost importance to ensure patient privacy and medical data protection. In general, through blockchain-based self-sovereign identity solutions patients are in control of their medical data and can grant and revoke access as needed. Privacy can be enhanced via zero-knowledge proofs, advanced cryptographic techniques, that verify the accuracy of data without revealing the data itself (Gaba et al., 2022). Moreover, to ascertain that sensitive information is protected from unauthorize access medical data can be stored encrypted, and authorized parties can only access it through cryptographic keys.

### DeSci in the longevity realm

Decentralized Science initiatives and organizations specifically focused on longevity are an exciting new vertical.

While many issues between general biomedical science and longevity science overlap, there are also distinct issues. As in general science, reproducibility is certainly a problem in longevity science, and longevity science will benefit from the endeavors of the DeSci community in making science more reproducible. Moreover, the incentives for the publishing and peer review process are also not aligned in the longevity sphere. However, compared to other biomedical fields such as cancer or Alzheimer’s research, longevity science and aging biology are severely underfunded. The risk averse behavior of traditional funding agencies is not set up to create the transformative science moonshots that will yield significant extensions in health- and lifespan. Hence, in the realm of Longevity, the DeSci movement has given birth to several Decentralized Autonomous Organizations that try to close this funding gap through the power of blockchain and token-based coordination mechanisms. Three notable Longevity DAOs that have already funded various projects, are VitaDAO, AthenaDAO, and CryoDAO. Each focus on different parts of the Longevity ecosystem but have in common that they provide innovative funding mechanisms and are community governed (Bischof et al., 2022; DeFrancesco and Klevecz, 2022; The community of the DAO, 2023; Fantaccini et al., 2024). VitaDAO focuses on commercializable longevity science, AthenaDAO focuses on women’s health research, and CryoDAO focuses on cryopreservation. Each utilizes blockchain technology for governance and funding decisions, allowing token holders to vote on which research projects to finance. Each also aims to democratize access to intellectual property generated from the research it funds.

Overall, decentralized science has the potential to significantly bolster longevity science by fostering a global, collaborative research ecosystem, where data and resources are coordinated across borders. This decentralized approach can accelerate the pace of discovery in longevity research, enabling more rapid development and validation of age-related interventions and therapies through community-driven funding and innovative, open-access platforms.

## Discussion

While the DeSci movement is still young, it is gaining momentum in several verticals. Big Pharma, traditional journals and academic institutions are taking notice of it. Moreover, DeSci meetings are happening across the globe bringing together people with different skillsets and experiences–creating diversity of thought, as DeSci is not just for scientists, but anyone who wants to accelerate science.

While DeSci promotes data transparency and traceability through blockchain as a means to improve reproducibility in science, other non-tech solutions may have similar effects. For example, Stewart and Plotkin argue that a stronger focus on the development of solid theoretical frameworks prior to empirical testing can suppress false positives in science and interacts synergistically with replication (Stewart and Plotkin, 2021).

Non-DeSci, community-driven and multi-governmental initiatives such as the European Open Access Cloud are developing data storage infrastructure to allow access to research data to all researchers that is not blockchain based (Budroni et al., 2019).

Moreover, transitioning to open access publishing is already accelerating due to the direct benefits of open publications to academics such as low-cost publishing, more citations, more media coverage, and transparent peer review (McKiernan et al., 2016). What DeSci adds here is another incentive through token-based compensation to the reviewers, as well as decentralized data storages.

DeSci DAOs are proliferating steadily, and the DeSci Wiki is counting 36 different DeSci DAOs as of May 2024 (DeSci Wiki, 2024). As of now most DeSci DAOs are focused on specific verticals of biotechnology. One reason for this might be, that in biotech it is still possible to create breakthrough science with rather small amounts of funding in the low millions, whereas other verticals of science and engineering are more costly. Another reason might be that in the blockchain world many people have a background in software, and often consider biology to be programable software.

However, for DeSci to become successful in the long run it must be able to fund many categories of science, and not be limited to certain ones. With the globally rising interest in cryptocurrencies, one could imagine a world where crypto becomes a formidable source of science funding and will in the future be able to compete with the R&D budget of nation states.

While DeSci has many advantages, there are several limitations to discuss. The new organization structure of a DAO–while being innovative and having certain benefits it also has difficulties to overcome.

While most DAOs intend to be decentralized, this probes to be a conundrum, especially in the early stages. At that stage, in many cases decentralization is merely a mirage, with the token distribution and hence the governance power being heavily pareto distributed and the DAO resembles more a Decentralized Autonomous Oligarchy, in which a minority of core contributors have the most governance power. However, the trend seems to be that over time tokens become more evenly distributed as more people are getting involved in the DAO. Furthermore, the ambiguity of the legal status of DAOs in the United States adds complexity, as there is no consensus or clear regulatory framework governing DAOs currently (Unlocking Scientific Innovation Through Decentralized, 2023).

That the first DeSci DAOs emerged in the Longevity space is not mere coincidence. The crypto community and the longevity community are both driven by unorthodox forward thinking, with the goal of creating a paradigm shift in their respective sectors. The first and most prominent crypto currency Bitcoin was created to decentralize money and disrupt the financial system. Longevity is disrupting the traditional medical system, moving the paradigm from current sick care to eternal healthcare. Additionally, as with every paradigm shift, both the idea of cryptocurrency, and the idea that aging is indeed malleable have initially been both faced harsh criticism and ridicule.

Moreover, the longevity field is open-minded when it comes to alternative funding mechanisms. They must, as funding for aging biology is scarce, with the 2023 yearly NIA budget dedicated to investigating the aging biology is only ∼$400M, compared to $7.3 billion for disease focused research such as cancer research (Fiscal Year 2024 Budget, 2024; NCI Budget and Appropriations, 2024). This environment provided a great entry point for the first DeSci DAO - VitaDAO–that paved the way for AthenaDAO, CryoDAO and other DAOs, with a community-first alternative funding model.

However, in recent years, due to the concern of the scientific community that the current funding landscape is mainly encouraging conservative and incremental progress in science, the traditional science world with its centralized institutions has also started promoting new initiatives and policies to foster high-risk high-reward research (OECD, 2021). Globally, this has led to the establishment of new funding schemes for high-risk high-reward research. However, like DAOs, these funding mechanisms and their results have not been evaluated rigorously due to their novelty (OECD, 2021).

While this article presents decentralized science as a solution to advance longevity research, there is no truly successful DeSci DAO yet. DAOs have shown themselves to be able to fund research and spin out companies, yet the ultimate verdict will be if they can indeed developed drugs and therapies that can slow, stall, or reverse the aging process. Moreover, it remains to be seen if DeSci DAOs can do this in a sustainable way by generating profits through successfully funded research and spinouts, and eventually finance expensive clinical trials. Time will tell but the DeSci adherents are increasingly rallying toward the cause.

## Data Availability

The original contributions presented in the study are included in the article/supplementary material, further inquiries can be directed to the corresponding author.
